# Development of in vitro airway epithelial model to assess immune response and safety of mucosal adjuvants

**DOI:** 10.1038/s41541-026-01459-z

**Published:** 2026-04-22

**Authors:** David Acosta, Mohammad Rubel Hoq, Kazuyo Takeda, Chad D. Costley, Hana Golding, Marina Zaitseva

**Affiliations:** 1 Division of Viral Products, Center for Biologics Evaluation and Research (CBER), Food and Drug Administration (FDA), Silver Spring, MD USA; 2 Microscopy and Imaging Core Facility, Center for Biologics Evaluation and Research (CBER), Food and Drug Administration (FDA), Silver Spring, MD USA; 3 BlueWillow Biologics, Inc., Ann Arbor, MI USA

**Keywords:** Diseases, Drug discovery, Immunology, Microbiology

## Abstract

Mucosal vaccines delivered intranasally or by inhalation, can elicit localized immunity that protects mucosal tissues at the site of infection and may reduce transmission of respiratory viruses, including respiratory syncytial virus (RSV), influenza virus, and SARS-CoV-2. Many mucosal vaccine candidates incorporate adjuvants to enhance and broaden immune response, underscoring the need for safe and effective formulations targeting the respiratory mucosa. Here, we employed differentiated human nasal and bronchial epithelial cells (hNEC and hBEC) cultured at air-liquid interface (ALI) to evaluate the soybean oil-in-water intranasal NE01 adjuvant, recently assessed clinically. Kinetic and dose-response studies showed that hBECs were more sensitive than hNEC to the cytopathic effect of NE01 as evidenced by loss of barrier integrity, reduced viability, and Interleukin-8 production. Spiking Cetylpyridinium chloride (CPC), a component of NE01, into a squalene oil-in-water adjuvant identified CPC as a potential contributor to the observed cytotoxicity in hBEC. Lower NE01 concentrations did not compromise hBEC viability or barrier function, indicating concentration-dependent effects and establishing thresholds for epithelial perturbation. The data provide proof-of-concept supporting the ALI system for assessing the effects of mucosal adjuvants on Upper and Lower respiratory tract epithelia during the early stages of development. This approach may help prioritize candidates while reducing reliance on animal use in preclinical evaluation.

## Introduction

Respiratory infections have a major impact on global health. Currently approved vaccines against influenza, respiratory syncytial virus (RSV) and severe acute respiratory syndrome coronavirus 2 (SARS-CoV-2) are all administered intramuscularly (parenterally) except for one intranasal flu vaccine (brand name FluMist)^1^. These intramuscular vaccines were shown to be effective in generating systemic immune responses that protect individuals from severe disease and hospitalization. However, parenteral vaccines are inefficient in eliciting immune responses at the mucosal-associated lymphoid tissue (MALT) of the respiratory tract, and, therefore, are inefficient in protecting the respiratory mucosa from infection. Recent studies indicated that while vaccinated populations generally clear the virus faster at early times post-infection, significant levels of infectious virus could be found in the nasopharynx of both vaccinated and unvaccinated individuals^2–4^.

Mucosal vaccines potentially present advantages over parenteral vaccines due to their ability to control viral infection at the upper respiratory tract (URT) through induction of secretory IgA antibodies within the mucosa^5^. High-affinity IgA provides a frontline of immune defense at mucosal surfaces, blocking and neutralizing toxins and pathogenic microbes^6,7^. Furthermore, the induced mucosal immune response might potentially curtail virus transmission from infected individuals^8^. There has been much interest in developing mucosal vaccines that induce both systemic and mucosal immunity against respiratory pathogens.

Mucosal vaccine development is constrained by the limited availability of adjuvants that can safely and effectively enhance nasal mucosal immune responses and promote induction of secretory IgA (sIgA) and systemic immunity^1,9,10^. The evaluation of novel mucosal vaccine adjuvants typically relies on extensive preclinical testing in animal models^11,12^. However, animal models have several limitations, including species-specific differences in immune responses that may limit translational relevance to humans^13,14^, as well as ethical and regulatory concerns associated with experimental animal use. Consequently, there is a growing imperative to replace animal-based preclinical testing of vaccines, including mucosal vaccines and adjuvants, with relevant in vitro models that accurately recapitulate the structural and functional characteristics of relevant human tissues.

Human peripheral blood mononuclear cells (PBMCs) and monocytes/macrophages, which are routinely used to assess the safety profile of parenteral adjuvants, are not appropriate to evaluate the safety of mucosal vaccines/adjuvants that target mucosal surfaces in the oral cavity, nose and airways, vagina, and small intestine. For testing of intranasal drug delivery in preclinical studies, the respiratory epithelial cell lines have been utilized, such as Calu-3 and RPMI 2650, that are easy to culture and demonstrate genetic homogeneity and unlimited lifespan. However, these models have limitations as they do not ciliate and differentiate appropriately compared to primary cultures^15,16^.

The air–liquid interface (ALI) model is established from primary human airway epithelial cells that are derived from nasal, proximal, or distal epithelium and are grown in cell cultures on a porous membrane support. The important characteristic of this model is the exposure of the apical side of cells to air, while the basal side of cells is in contact with nutrients provided in the medium on the basal side of the membrane. Under ALI conditions, cell cultures differentiate into a pseudostratified epithelium in about 3–5 weeks, with basal cells, cilia projecting from the apical surface, and goblet cells producing mucus^17,18^. Human ALI epithelial cell cultures are very similar to those cells of the epithelial lining of human airways^19,20^. As a physiologically relevant platform, ALI models were used to study mucosal epithelial function and pathology associated with infections with respiratory viruses^21–27^. However, the potential of an in vitro airway epithelial cell model to assess and predict the safety of mucosal vaccines/adjuvants has not been investigated.

NE01 is an oil-in-water emulsion containing soybean oil, Tween 80, and the cationic surfactant, cetylpyridinium chloride (CPC) and is designed to function as an intranasal adjuvant. NE01 has been found to be safe in multiple animal species and humans^28–30^. For example, this adjuvant has been shown to improve antibody responses following intranasal vaccination of mice with inactivated pandemic H1N1 influenza vaccine^31^, to produce strong cellular and humoral responses in ferrets and protect them from lethal challenge with H5N1 influenza virus^32,33^. In a recently completed clinical trial (NCT05397119), an H5N1 vaccine adjuvanted with NE01 adjuvant (W_80_5EC) was well tolerated and demonstrated strong cross-clade immune system priming of mucosal (IgA/IgG), systemic (Hemagglutination-Inhibition and antibody-dependent cellular cytotoxicity), and cellular (memory B cell and CD4^+^ T cell) immune responses^34^.

In this study, ALI cultures of primary human bronchial epithelial cells (hBEC) and nasal epithelial cells (hNEC) were employed to evaluate the effects of NE01 adjuvant on respiratory epithelium, including cell viability, tissue/barrier integrity, and cytokine production. Here, we describe a human airway epithelial model that meaningfully supports the principles of Replacement, Reduction, and Refinement (3Rs). This approach contributes to ongoing efforts to reduce animal use in vaccine development and provides a sustainable, human-relevant platform for the evaluation of mucosal vaccination strategies, which are of increasing interest.

## Results

### Treatment of hBECs with NE01 emulsion

FluMist is administered via a standard sprayer that deposits vaccine primarily on the nasal and upper airway mucosa. Emerging vaccine platforms—especially aerosolized or finely nebulized intranasal vaccines—are designed to reach deeper into the bronchi and lower respiratory tract (LRT) to stimulate mucosal immunity there^35–37^. While this can enhance protective immunity, it also introduces distinct safety concerns. The lower airways (bronchi, bronchioles, and lung parenchyma) have more sensitive and reactive tissues than the nasal passages, with a greater risk for irritation, inflammation, and injury if particles or formulation components are deposited there at high concentration^38,39^.

To assess whether human airway epithelial cells can serve as a model for evaluating the effects of mucosal adjuvants on the LRT epithelium, primary hBECs obtained from healthy adult donors were cultured on porous membrane supports for 28 days under ALI conditions. NE01 nanoemulsion adjuvant, a 60% formulation of the W_80_5EC nanoemulsion (60% W_80_5EC) containing soybean oil, Tween 80, CPC, and ethanol in water, was used for cell treatments as a model adjuvant^28^. The volume of NE01 adjuvant was adjusted based on the approximately 300 to 600 ratio between the surface area of the human nasal cavity (~90 or 181 cm²) and the growth area of the cell culture inserts (0.33 cm²)^40,41^. Based on this ratio, the 500 µL dose of NE01-adjuvanted vaccine administered to humans^34^ was scaled to 1.0 µL and applied in 100 µL of Complete Basal Medium to ensure full apical surface coverage of the cell cultures. Because the NE01 stock formulation (60% W_80_5EC) contains approximately 40% soybean oil, this 1:100 dilution resulted in a final oil concentration of 0.4%. Cell cultures were exposed to the NE01 adjuvant via the apical surface.

**Fig. 1:**
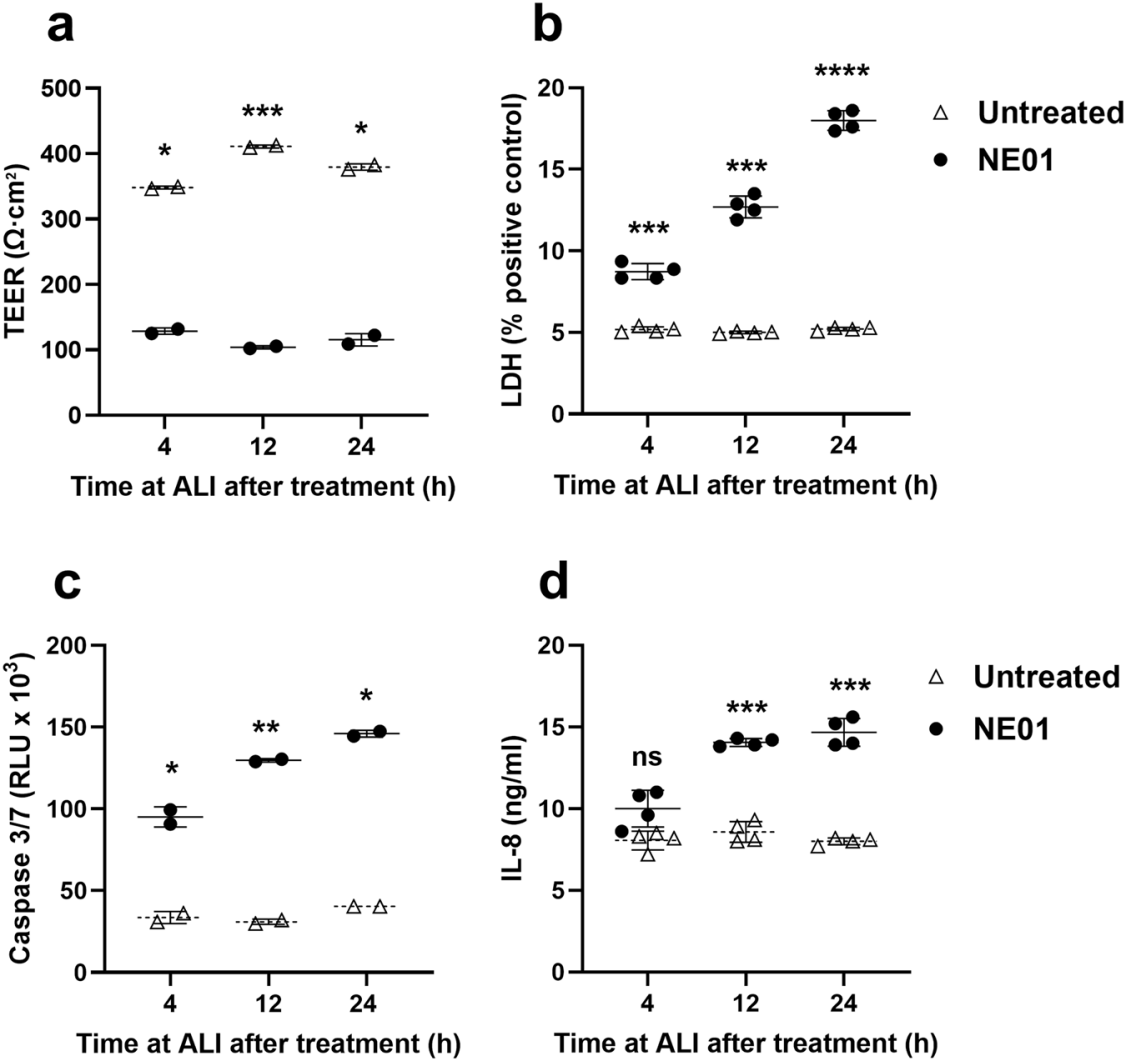
Kinetics of changes in barrier integrity and IL-8 cytokine production in hBECs in response to treatment with NE01 adjuvant. hBECs (duplicate cell cultures from a single donor) were treated apically with NE01 emulsion (0.4% oil) for 1 h, after which NE01 was removed, and the cells were returned to air–liquid interface (ALI) conditions. At 4-, 12-, and 24-h post treatment, epithelial barrier integrity was assessed by measuring TEER (**a**) and basal supernatants and lysates from membrane-attached cells were collected to evaluate cytotoxicity via lactate dehydrogenase (LDH) release using the CytoTox 96 non-radioactive cytotoxicity assay (**b**), apoptosis via Caspase 3/7 activity using the Caspase-Glo 3/7 3D kit (**c**), and IL-8 secretion by ELISA (**d**). Data were presented as scatter dot plots with mean ± SD. For TEER and Caspase 3/7, each dot represents an individual cell culture (*n* = 2), and error bars indicate variability between duplicate cultures. For LDH and IL-8, each dot represents a technical replicate (two per culture, two cultures, total *n* = 4), and error bars reflect variability among technical replicates. Data were representative of two experiments using hBECs from individual donors. ****P ≤ 0.0001, ***P ≤ 0.001, **P ≤ 0.01, and *P ≤ 0.05 by two-way ANOVA with Sidak’s multiple comparison test.

To elucidate the kinetics of hBEC response to the NE01 adjuvant, hBECs were left untreated or were incubated with NE01 adjuvant (0.4%) for 1 h. The adjuvant was then removed, and cell cultures remained at ALI for 4-, 12-, or 24-h. At the end of the culture period, tissue integrity was assessed by measuring transepithelial electrical resistance (TEER) and cell apoptosis by measuring Caspase 3/7. Cell culture medium in the basolateral chamber (basal medium) was analyzed for lactate dehydrogenase (LDH) release, a marker of cell death, and interleukin-8 (IL-8) secretion (Fig. 1). In untreated hBEC, TEER ranged between 350 to 400 Ω·cm² during observation period, as expected for well-differentiated hBECs that form a strong epithelial barrier (Fig. 1a). In NE01-treated cells, TEER was significantly reduced to around 130 Ω·cm² at 4-h post-exposure (a 70% decrease) and remained at similarly low levels at 24-h (Fig. 1a). LDH in the basal medium increased about 50% in NE01-treated cells compared with untreated cells at 4-h post-exposure and further increased by 2.5- and 4-fold above untreated cells at 12- and 24-h, respectively (Fig. 1b). To study whether NE01 compromises respiratory epithelial cell barrier by inducing cell apoptosis, the Caspase 3/7 serine protease activity was measured in cell lysates. Activation of Caspase 3/7 was evident at 4 h and continued to increase at the 12- and 24-h timepoints (Fig. 1c). These data indicated that treatment of hBECs with NE01 at 0.4% oil resulted in reduced tissue integrity, as demonstrated by significant loss of TEER, followed by Caspase 3/7 activation and increased LDH release indicative of reduced cell viability. hBECs are known to constitutively produce the inflammatory cytokine IL-8^42^. At 4 h, similar levels of IL-8 were detected in the basal medium of both NE01-treated and untreated cells. However, at 12- and 24-h post-exposure, IL-8 levels were significantly increased in NE01-treated cells compared with untreated controls (Fig. 1d). Based on the kinetic analyses, the 12-h time point was selected for subsequent experiments, as it demonstrated a dynamic range in LDH release, Caspase 3/7 activity, and IL-8 production.

**Fig. 2:**
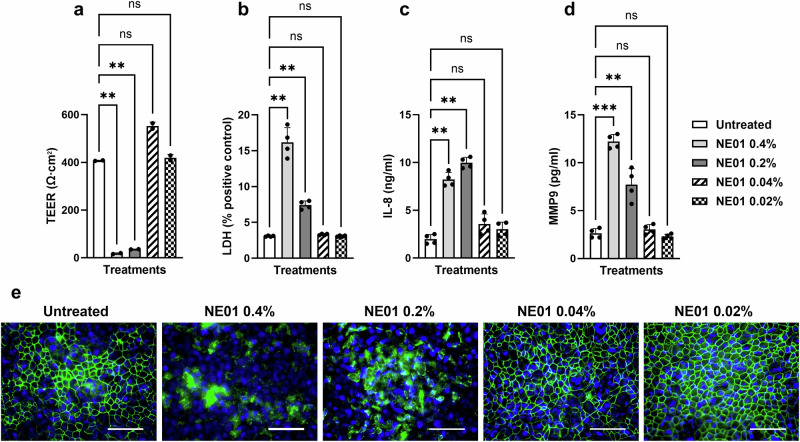
NE01 dose-response in hBECs. hBECs (duplicate cell cultures from a single donor) were treated apically with NE01 emulsion dilutions containing 0.4, 0.2, 0.04, or 0.02% oil for 1 h after which NE01 dilutions were removed, and cell cultures were returned to ALI. At 12-h post treatment, TEER (**a**), LDH release (**b**), and IL-8 (**c**) were measured as described in the legend to Fig. 1, and MMP9 (**d**) were measured in basal supernatants by ELISA. Data were presented as scatter dot plots with mean ± SD. For TEER and Caspase 3/7, each dot represents an individual cell culture (*n* = 2), and error bars indicate variability between duplicate cultures. For LDH, IL-8, and MMP9, each dot represents a technical replicate (two per culture, two cultures, total *n* = 4), and error bars reflect variability among technical replicates. Data were representative of two experiments using hBECs from individual donors. ***P ≤ 0.001 and **P ≤ 0.01 by one-way ANOVA with Dunnett’s multiple comparison test. **e** Representative immunofluorescent images show the expression of Zonula occludens-1 (ZO-1) protein (green) in hBECs following treatment with NE01 adjuvant dilutions. Nuclei were stained with DAPI (blue). Scale bar, 50 µm. Note loss of ZO-1 membrane expression in hBECs treated with NE01 0.4% and NE01 0.2% oil that correlated with the loss of barrier integrity (**a**) and reduced viability (**b**).

Next, hBECs were exposed to NE01 diluted to contain a range of oil concentrations, from 0.4 to 0.02% oil (Fig. 2a–d). NE01 at 0.4% and 0.2% significantly reduced TEER and increased LDH levels in treated hBECs. In contrast, at lower concentrations (0.04 and 0.02% oil), NE01 did not compromise barrier function or induce cell death (Fig. 2a, b). In addition to markers of tissue integrity and inflammation, the levels of Matrix Metalloprotease-9 enzyme (MMP9), known to play a role in remodeling the extracellular matrix under physiological and pathological conditions, was assessed in the basal medium^43,44^. A three- to four-fold increase in IL-8 and MMP9 production was measured in hBECs treated with NE01 at 0.4 and 0.2% oil compared with untreated cells. At the same time, there were no differences in the levels of IL-8 and of MMP9 in hBECs treated with NE01 at low concentrations (0.04 and 0.02% oil) compared with untreated cells (Fig. 2c, d, respectively).

To visualize the effects of NE01 treatment on the structural integrity of the respiratory epithelium, NE01-treated and untreated hBECs were fixed and stained with antibodies against Zonula Occludens-1 (ZO-1), a marker of Tight Junctions (TJ) that support cell-to-cell adhesion in the epithelial barrier. In untreated cells, strong membrane expression of ZO-1 was noted (Fig. 2e). In hBECs treated with NE01 at 0.4 and 0.2% oil, membrane localization of TJ protein ZO-1 was lost, indicating disruption of TJ. In contrast, at lower NE01 concentrations (0.04 and 0.02%), TJ remained intact as evidenced by strong membrane-associated ZO-1 expression comparable to that of the untreated control (Fig. 2e). These observations were consistent with findings from the other two assays used to assess respiratory epithelial integrity in the hBEC model.

**Fig. 3:**
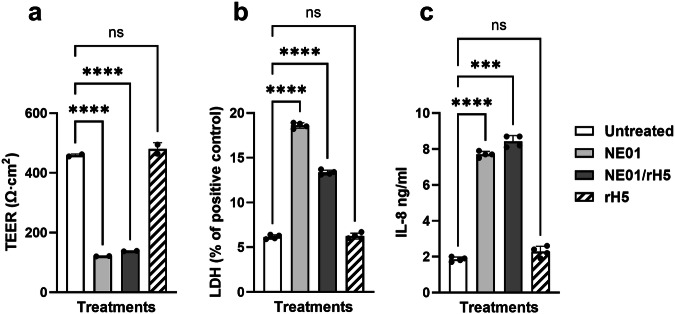
rH5 protein does not attenuate the effect of NE01 adjuvant on hBECs. hBECs were exposed to NE01 alone, NE01 formulated with rH5, or rH5 alone via the apical surface for 1 h, after which all treatments were removed, and cell cultures were returned to ALI. At 12-h post treatment, TEER (**a**) LDH release (**b**) and IL-8 (**c**) were assayed as described in the legend to Fig. 1. Note that rH5 alone did not show any effect on barrier integrity, cell viability, or inflammatory response. All treatments were performed in duplicate cell cultures from a single donor. Data were presented as scatter dot plots with mean ± SD. For TEER, each dot represents an individual cell culture (*n* = 2), and error bars indicate variability between duplicate cultures. For LDH and IL-8, each dot represents a technical replicate (two per culture, two cultures, total *n* = 4), and error bars reflect variability among technical replicates. Data were representative of two experiments using hBECs from individual donors. ****P ≤ 0.0001 and ***P ≤ 0.001 by one-way ANOVA with Dunnett’s multiple comparison test.

In the next experiment, we sought to determine whether formulation of NE01 adjuvant with a vaccine antigen, such as recombinant influenza A virus hemagglutinin (rH5), attenuates the effect of NE01 on hBECs. Vaccine formulations were prepared by mixing one volume of NE01 diluted to 0.4% oil with two volumes of appropriately diluted rH5 in formulation buffer (Fig. 3). The results showed that rH5 alone (0.6 µg per cell culture) did not reduce TEER or cell viability and did not increase production of IL-8 (Fig. 3a–c, respectively). In contrast, combining rH5 with NE01 at 0.4% oil resulted in similar levels of TEER loss, cell death, and IL-8 secretion as NE01 alone (Fig. 3 vs. 2). Thus, rH5 did not attenuate the effect of NE01 on cell viability, TEER, or IL-8 production (Fig. 3b vs. 3a, c).

### Role of CPC in the disruption of epithelial integrity

**Fig. 4:**
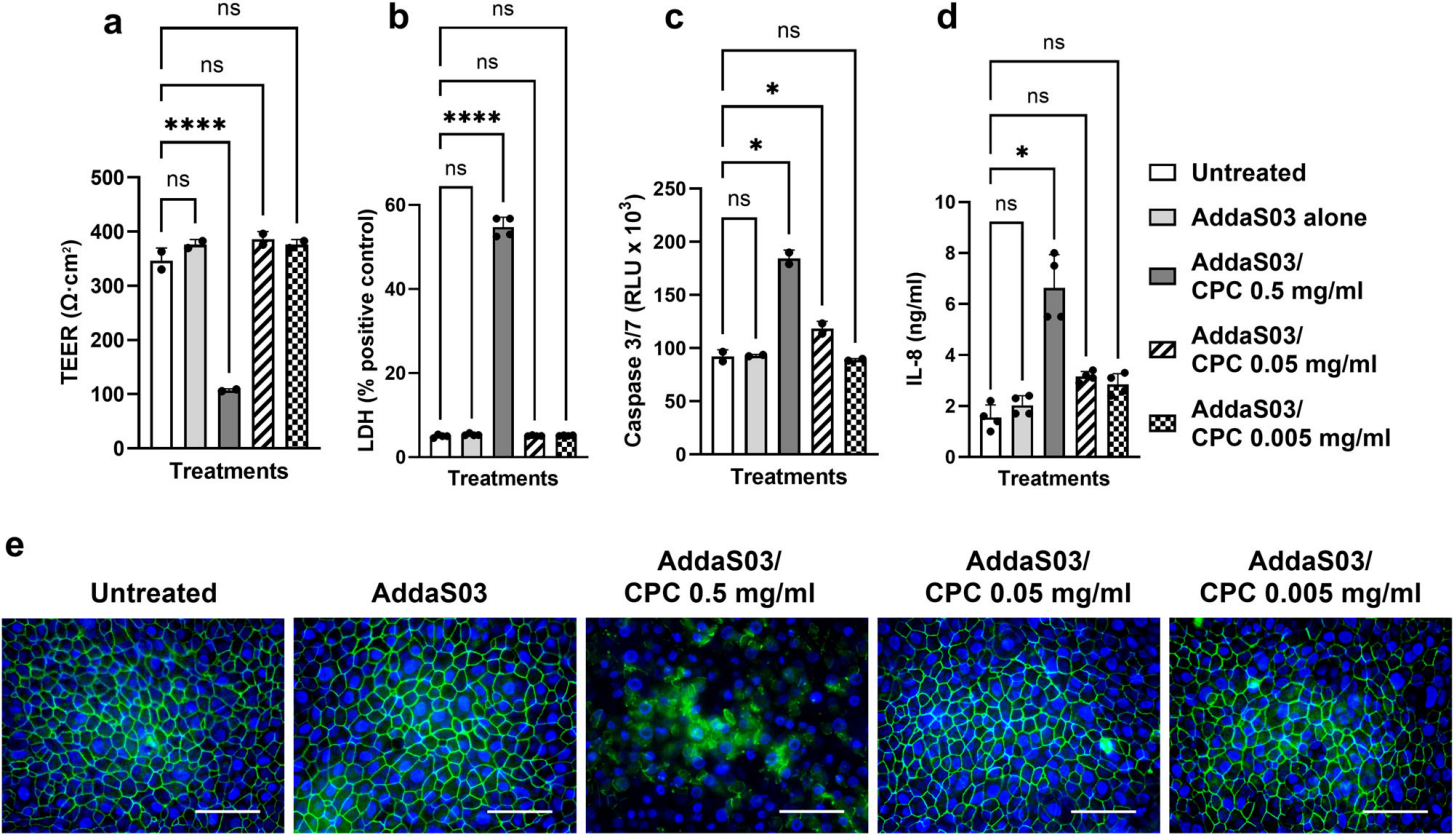
Squalene oil-in-water adjuvant induced loss of epithelial cell barrier and inflammatory response in hBECs only in the presence of CPC. hBECs (duplicate cell cultures from a single donor) were treated with AddaS03 adjuvant alone or with AddaS03 with CPC at 0.5, 0.05, or 0.005 mg/mL apically for 1 h, after which all treatments were removed, and cell cultures were returned to ALI. At 12-h post treatment, the effect of AddaS03 alone or AddaS03 with CPC on the epithelial cell integrity, viability, and inflammation were assayed by TEER (**a**) LDH release (**b**) Caspase 3/7 (**c**) and IL-8 (**d**) as described in the legend to Fig. 1. Data were presented as scatter dot plots with mean ± SD. For TEER and Caspase 3/7, each dot represents an individual cell culture (*n* = 2), and error bars indicate variability between duplicate cultures. For LDH and IL-8, each dot represents a technical replicate (two per culture, two cultures, total *n* = 4), and error bars reflect variability among technical replicates. Data are representative of two experiments using hBECs from individual donors. ****P ≤ 0.0001 and *P ≤ 0.05 by one-way ANOVA with Dunnett’s multiple comparison test. **e** Representative immunofluorescent images show strong membrane ZO-1 protein expression (green) in hBEC treated with AddaS03 adjuvant alone and complete loss of membrane ZO-1 in hBEC treated with AddaS03 with CPC 0.5 mg/ml. Nuclei were stained with DAPI (blue). Scale bar, 50 µm.

NE01 nanoemulsion adjuvant contains CPC, a cationic surfactant used to stabilize nano-sized droplets during homogenization. In nonclinical and in clinical studies, NE01 has been mixed with vaccine candidates to achieve final NE01 concentrations ranging from 5 to 40% (v/v) (corresponding to 0.5 to 4 mg/mL CPC) and 20% (v/v) (CPC at 2 mg/mL), respectively^28,34^. To determine whether the observed loss of TEER, reduced cell viability, and increased inflammatory cytokine production in NE01-treated hBECs are mediated by the oil or CPC, we used the commercial oil-in-water adjuvant AddaS03 containing 5% (v/v) squalene oil and α-tocopherol as a surrogate model of NE01 formulated without CPC. AddaS03 alone diluted to 0.4% oil (to match oil concentration in NE01 used for cell treatments) or AddaS03 spiked with CPC at 0.5 mg/mL was added to the apical surface of hBEC. In addition, a CPC dose-response was evaluated by spiking AddaS03 with CPC at 0.05 and 0.005 mg/mL. After 1-h incubation, the adjuvant mixtures were removed from the apical surface, and hBECs were maintained under ALI for 12-h (Fig. 4). The results showed that AddaS03 adjuvant alone, diluted to the same oil concentration as NE01 (0.4%), did not reduce TEER or cell viability and did not increase Caspase 3/7 or IL-8 compared with untreated cells (Fig. 4a–d). In contrast, hBECs treated with AddaS03 spiked with CPC at 0.5 mg/mL, exhibited a significant reduction in TEER accompanied by an increase in LDH release, Caspase 3/7 activity, and IL-8 production. Lower CPC concentrations (0.05 and 0.005 mg/mL) did not disrupt epithelial integrity or increase IL-8 levels although a modest increase in Caspase 3/7 activity was observed at 0.05 mg/mL (Fig. 4c). To confirm the dose-dependent effects of CPC, fixed hBECs were stained with anti-ZO-1 antibodies (Fig. 4e). Strong membrane localization of ZO-1 was observed in both untreated cells and in cells treated with AddaS03 alone, indicating that squalene oil emulsion did not alter ZO-1 distribution. In contrast, CPC at 0.5 mg/mL caused loss of membrane ZO-1, suggesting disruption of TJ. Lower CPC concentrations (0.05 and 0.005 mg/mL) did not affect ZO-1 localization, consistent with the results of the other assays (Fig. 4). Together, these findings indicate that the CPC component of NE01 adjuvant may contribute to the loss of respiratory epithelial integrity and increase in IL-8 production in a dose-dependent manner.

### Nasal epithelial cells are partially resistant to the disruptive effect of NE01

**Fig. 5:**
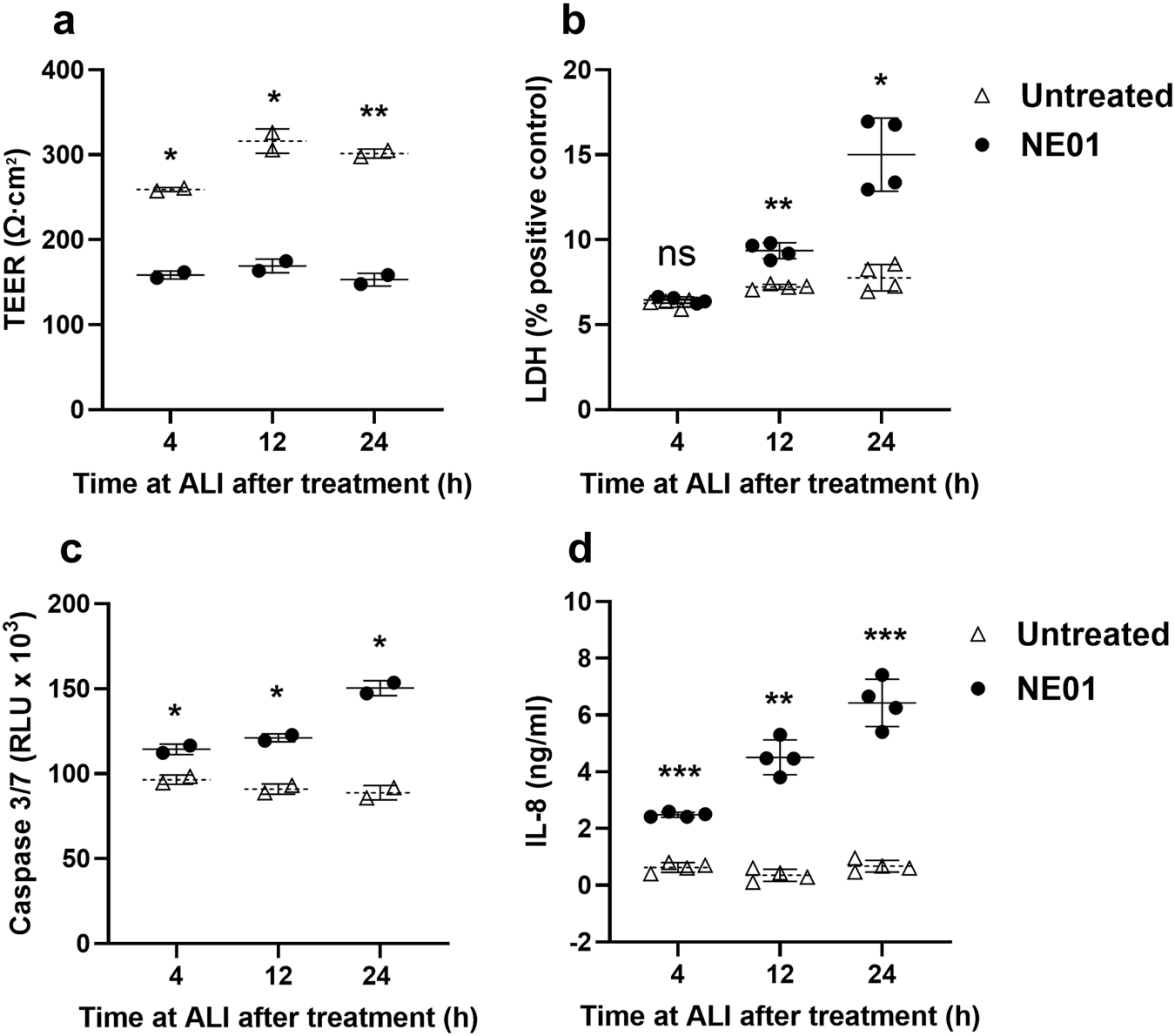
Kinetics of changes in barrier integrity and IL-8 cytokine production in hNECs in response to treatment with NE01 adjuvant. hNECs (duplicate cell cultures from a single donor) were treated apically with NE01 emulsion (0.4% oil) for one hour, after which NE01 was removed, and the cells were returned to air–liquid interface (ALI) conditions. At 4-, 12-, and 24-h post treatment, TEER (**a**) LDH release (**b**) Caspase 3/7 (**c**) and IL-8 (**d**) were measured as described in the legend to Fig. 1. Data were presented as scatter dot plots with mean ± SD. For TEER and Caspase 3/7, each dot represents an individual cell culture (*n* = 2), and error bars indicate variability between duplicate cultures. For LDH and IL-8, each dot represents a technical replicate (two per culture, two cultures, total *n* = 4), and error bars reflect variability among technical replicates. Data were representative of two experiments using hNECs from individual donors. ***P ≤ 0.001, **P ≤ 0.01, and *P ≤ 0.05 by two-way ANOVA with Sidak’s multiple comparison test.

Because some mucosal vaccine applications are restricted to the nasal cavity, it was important to determine whether hNECs are sensitive to NE01. hNEC were differentiated for 28 days and then treated with NE01 adjuvant (0.4% oil) for 1 h. After removal of the adjuvant, cultures were returned to ALI, and responses were evaluated at 4-, 12-, and 24-h (Fig. 5). In untreated hBEC, TEER ranged from 250 to 320 Ω·cm² over the observation period, which was slightly lower than that observed in untreated hBEC, consistent with previous reports^45^. Following NE01 treatment, TEER in hNEC decreased to approximately 150 Ω·cm² at 4-h post-exposure (∼40% reduction compared to untreated cells) and remained at similar levels at 12- and 24-h post treatment (Fig. 5a). Cell viability (LDH) was similar between untreated and treated cells at 4-h post treatment but LDH levels increased approximately 20% and 2-fold at 12- and 24-h, respectively (Fig. 5b). Caspase 3/7 activity increased by 20 to 30% at 4- and 12-hours post treatment and continued to rise at 24-h (Fig. 5c). IL-8 levels were elevated in NE01-treated hNECs at 4-h compared with untreated cells and continued to increase at 12- and 24-h (Fig. 5d). Overall, the response to NE01 treatment was somewhat slower in hNEC than in hBEC, with a smaller reduction in TEER and better preservation of cell viability observed in hNEC.

**Fig. 6:**
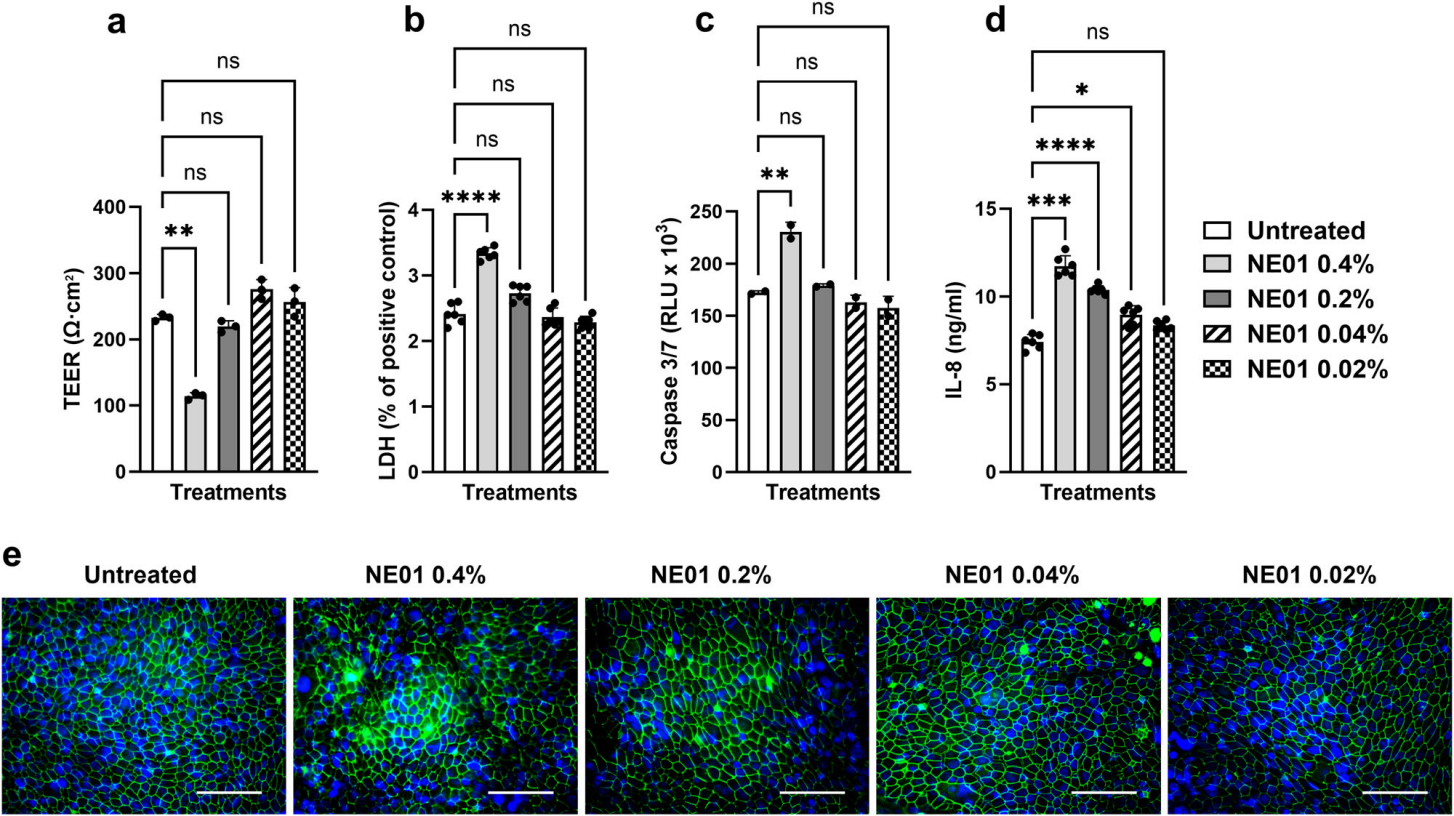
NE01 dose-response in hNECs. hNECs (triplicate cell cultures from a single donor) were treated apically with NE01 adjuvant dilutions containing 0.4, 0.2, 0.04, or 0.02% oil for 1 h after which NE01 dilutions were removed, and cell cultures were returned to ALI. At 12-h post treatment, TEER (**a**) LDH (**b**) Caspase 3/7 (**c**) and IL-8 (**d**) were measured as described in the legend to Fig. 1. Data were presented as scatter dot plots with mean ± SD. For TEER and Caspase 3/7, each dot represents an individual cell culture (*n* = 3 and *n* = 2, respectively), and error bars indicate variability between duplicate and triplicate cultures, respectively. For LDH and IL-8, each dot represents a technical replicate (two per culture, three cultures, total n = *6*), and error bars reflect variability among technical replicates. Data were representative of two experiments using hNECs from individual donors. ****P ≤ 0.0001, ***P ≤ 0.001, **P ≤ 0.01, *P ≤ 0.05 by one-way ANOVA with Dunnett’s multiple comparison test. **e** Representative immunofluorescent images show the expression of ZO-1 protein (green) in hNECs following treatment with NE01 adjuvant dilutions. Nuclei were stained with DAPI (blue). Note only minor loss of ZO-1 membrane expression in hNECs treated with NE01 0.4% compared with untreated cells and strong membrane ZO-1 expression in hNEC following treatment with NE01 at 0.2% oil that correlated with preserved membrane integrity (**a**) and cell viability (**b**). Scale bar, 50 µm.

In a dose-response study, differentiated hNEC were incubated with apically applied NE01 adjuvant for 1 h followed by incubation at ALI conditions for 12 h (Fig. 6). In untreated hNECs, TEER was ~230 Ω·cm². Treatment with NE01 (0.4% oil) reduced TEER by ∼50% to about 115 Ω·cm², whereas at lower NE01 concentrations, TEER remained comparable to untreated controls (Fig. 6a). LDH release increased by ∼30% in hNECs exposed to NE01 at 0.4% oil, consistent with a ∼30% increase in Caspase 3/7 activity (Fig. 6b, c). No increase in LDH or in Caspase 3/7 was observed at lower NE01 concentrations. IL-8 levels were modestly elevated in NE01-treated hNEC in a dose-dependent manner (Fig. 6d). A slight reduction in membrane ZO-1 expression was observed in hNECs treated with 0.4% NE01, but not at lower concentrations (0.04 and 0.02%) (Fig. 6e). Overall, these data indicate that NE01 induces only modest disruption of barrier integrity in hNEC at the highest concentration tested without substantial loss of cell viability. These findings suggested that hNEC may be more resistant to NE01 than hBEC (Figs. 2 vs. 6)

### Comparison of epithelial architecture in ALI-differentiated hNEC and hBEC cultures

**Fig. 7:**
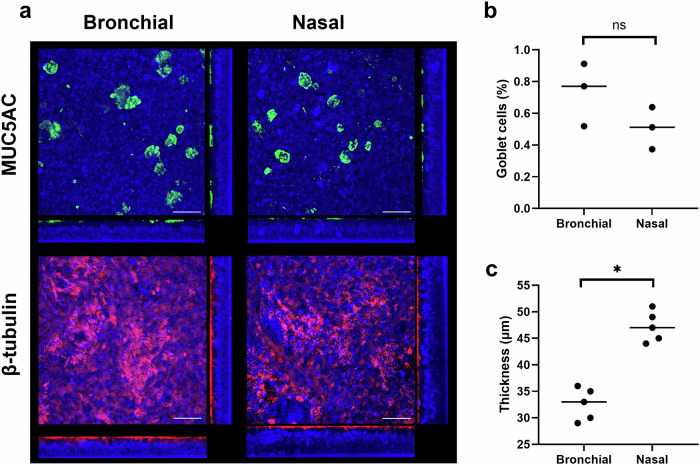
Frequency of MUC5AC-positive cells and cilia expression in differentiated hBEC and hNEC. Representative confocal microscopy images of ALI-differentiated human nasal and bronchial epithelial cells stained for MUC5AC (green) and β-tubulin (red), with nuclei counterstained with DAPI (blue), acquired as z-stacks (**a**). Scale bar, 50 µm. Top panels show En face (surface XY) views of MUC5AC-positive goblet cells; lower panels show En face views of β-tubulin–positive ciliated cells. Inserts on the bottom and right of each panel display orthogonal (XZ and YZ) projections, demonstrating apical localization of MUC5AC expression and epithelial thickness defined by cilia. MUC5AC-positive cells were quantified and normalized to the number of nuclei per microscopic field (*n* = 3) acquired from a single donor per cell type (Supplementary Table 1) (**b**). Average epithelial thickness in hBEC and hNEC stained with anti-β-tubulin antibodies was measured from 3D reconstructions of five images per cell type (**c**). 3D reconstruction, thickness measurements, nuclear counting, and intensity measurement were performed using Imaris software. Data are presented as scatter dot plots with mean ± SD; each dot represents an individual microscopic field, and error bars indicate variability among technical replicates (*n* = 3 and *n* = 5 in **b** and **c**, respectively). Graphs were generated using Graphpad Prism (Dotmatics). Data were representative of two independent experiments using hBECs and hNECs derived from individual donors. *P ≤ 0.05 by unpaired, two-tailed nonparametric Mann–Whitney *U*-test; ns not significant.

To assess whether differences in susceptibility to NE01-mediated epithelial barrier disruption are associated with structural differences between airway epithelial models, ALI-differentiated hBECs and hNECs from non-paired donors were stained with antibodies against MUC5AC, a marker of goblet cells, and β-tubulin, a marker of cilia (Fig. 7). Discrete MUC5AC-positive cells were distributed across the epithelial surface of both hBEC and hNEC cultures (Fig. 7a, top panels), with orthogonal projections confirming apical localization of MUC5AC expression. Quantitative analysis demonstrated no significant difference in the frequency of MUC5AC-positive goblet cells between hBECs and hNECs (Fig. 7b and Supplementary Table 1).

β-tubulin staining revealed dense and uniform ciliation across the epithelial surface in both epithelial models, consistent with extensive ciliary differentiation (Fig. 7a, lower panels). Orthogonal projections demonstrated a continuous ciliary layer at the apical surface, indicative of well-differentiated epithelium in both hBEC and hNEC cultures (orthogonal x/z and y/z views in Fig. 7a lower panels). Average epithelial thickness was determined from 3D reconstructions of five images per cell type and was greater in hNEC cultures than in hBEC cultures (Fig. 7c). Together, these data indicate that ALI-differentiated hBEC and hNEC cultures exhibit comparable goblet cell frequencies and robust ciliation, while increased epithelial thickness in hNECs may contribute to their reduced sensitivity to NE01-induced barrier disruption.

## Discussion

This study aimed to assess whether primary human nasal and bronchial epithelial cell cultures can serve as surrogate in vitro models for evaluating responses to mucosal adjuvants. ALI-differentiated bronchial epithelial cells exhibited marked disruption of epithelial barrier integrity and reduced viability following exposure to the soybean oil-in-water mucosal adjuvant NE01. Importantly, cytopathic effects were not observed at lower NE01 concentrations, indicating a clear concentration-dependent response in hBECs. These findings suggest the possibility of a threshold level of NE01 exposure above which epithelial perturbation may become detectable, a consideration that may be relevant when interpreting in vitro observations in the context of mucosal vaccine formulations. Experiments using the squalene-based oil-in-water emulsion AddaS03 alone or in combination with the CPC surfactant present in the NE01 formulation, indicate that CPC contributes to the impairment of barrier function and cell viability in human bronchial epithelial cells. In contrast, alterations in epithelial barrier function were less pronounced in nasal epithelial cells. Together, these findings support the complementary use of nasal and bronchial epithelial models as in vitro tools for early-stage evaluation and initial screening of novel mucosal adjuvants.

The airway epithelium forms a physical barrier that prevents foreign particles and pathogens from entering the submucosal compartment. The continuous sheet of epithelial cells comprises different cell types, including ciliated, goblet, basal, and club cells. The integrity of this barrier relies on TJ, which are intracellular connections between adjacent epithelial cells^46,47^. TJ are the most apical junctional complexes and regulate the selective passage of ions and solutes through the paracellular space, while preventing the migration of pathogens from the lumen into the interstitium. More than 40 proteins are involved in TJ structure and function, including transmembrane proteins such as occludens (OCLN) and claudins (CLD) that form intracellular adhesions and scaffold proteins Zonula Occludens (ZO)-1, ZO-2, and ZO-3 that link OCLN and CLDs to the actin cytoskeleton^48,49^. In healthy epithelial tissues, ZO-1 is localized exclusively at the TJ. In hBECs exposed to NE01, the ZO-1 protein levels were significantly reduced and dislocated away from the cell membrane, which correlated with a marked decrease in TEER. The concurrent reduction in ZO-1 membrane-associated ZO-1 and loss of TEER in NE01-treated hBECs is consistent with previous studies showing that mammary epithelial cells depleted of ZO-1 were unable to maintain epithelial resistance^50^. Although the precise mechanism underlying the loss of transepithelial resistance (i.e., increased conductance) and the disruption of ZO-1 membrane localization in NE01-treated hBECs was not directly investigated in this study, the concurrent increase in MMP9 levels observed in the same cultures suggests a potential mechanistic link. Specifically, MMP9 may contribute to TJ disruption, reduced cell viability, and the associated decline in TEER, consistent with prior reports in human airway epithelial models^51^.

In addition to disrupting barrier function, hBECs cultures exposed to NE01 exhibited increased LDH release, indicative of cell death, along with elevated Caspase 3/7 activity, suggesting that the NE01 emulsion at 0.4% oil induces apoptosis in bronchial epithelial cells following a 1-h exposure. Our findings in hBECs extend and confirm earlier observations of apoptosis of TC-1 mouse epithelial cells exposed to the NE01 adjuvant^52^. Notably, the same study demonstrated that the antigen-presenting dendritic cells (DC) can engulf apoptotic, antigen-loaded mouse epithelial cells. This indirect route of antigen acquisition promoted DC maturation and was proposed to play a key role in the adjuvant properties of NE01^52^. Therefore, NE01-induced disruption of the epithelial barrier and associated epithelial cell apoptosis, may enhance paracellular permeability and facilitate the translocation of antigen-loaded epithelial cells, thereby contributing to DC priming in the MALT underlying the respiratory epithelial barrier.

Earlier animal studies used vaccine formulations containing NE01 at concentrations ranging from 0.1 to 2%^53,54^, followed by investigations evaluating higher concentrations between 5 and 40%^28^. In both, preclinical and clinical studies, NE01 was admixed with vaccine antigen at a 1:2 ratio, corresponding to a one-third of the stock, or 20% NE01 adjuvant^32–34,55^. In the present study, NE01 concentrations that induced epithelial barrier perturbation in hBECs, 0.4 oil and 0.2% oil, were approximately threefold and 1.5-fold higher, respectively, than those used in preclinical and clinical vaccine formulations. In contrast, lower concentrations (0.04 and 0.02%) did not produce detectable cytopathic effects in hBEC cultures (Fig. 2). These dose-dependent effects observed in hBECs support the existence of a threshold concentration beyond which epithelial perturbation may occur.

In search for the component of NE01 responsible for apoptosis in hBECs, we performed spiking experiments with the cationic surfactant CPC, a quaternary ammonium compound (QAC), which is used as an antimicrobial agent^56^. Studies in animal models have shown that CPC improves the affinity of the NE01 adjuvant to mucin, leading to increased retention of antigen at the application site and resulting in increased immunogenicity of an NE01-adjuvanted vaccine^57^. Thus, the inclusion of CPC in the adjuvant formulation prolongs nasal residence, allowing it to exceed the 15–20-min mucociliary clearance half-life reported for liquid and powdered formulations^58^. The 1-h exposure of cell cultures to NE01 adjuvant that was used in our experiments in vitro aligned with the expected duration of exposure to intranasally administered NE01-adjuvanted vaccine in humans.

The CPC concentrations in NE01-adjuvanted vaccine formulations varied from CPC at 0.01−0.2 mg/mL in NE01 0.1 to 2% to 0.5−4 mg/mL of CPC in 5 to 40% NE01 and CPC at 2 mg/mL in 20% NE01^28,32–34,53–55^. In our experiments, AddaS03 adjuvant containing squalene oil (AddaS03 adjuvant is similar in composition to AS03 adjuvant included in H5N1 pandemic influenza vaccine Arepanrix developed at GSK and licensed in Europe, Canada, and USA) was used as a surrogate model of NE01 formulated without CPC. AddaS03 alone did not have any cytotoxic effect on hBECs when used at the same oil content as NE01. For spiking experiments, CPC was used at 0.5 mg/mL, a concentration within the range employed in nonclinical and clinical studies. The results showed that AddaS03 spiked with CPC at 0.5 mg/mL but not at lower concentrations reduced TEER and cell viability, which correlated with increased activation of Caspase 3/7 in hBEC cultures. Although the experimental conditions did not allow comparison of NE01 formulated with and without CPC, the spiking experiment results suggest that CPC may contribute to the loss of barrier integrity in hBECs following treatment with NE01,consistent with reports that showed increased apoptosis in A549 lung epithelial cells exposed to CPC^59^. Cationic surfactants from the QAC group, have been reported to disrupt microbial membrane integrity^60^, facilitate protein denaturation^61^, and impair mucociliary clearance^62^. In vivo, QAC exposure has been associated with pulmonary inflammation in mice and rats^63,64^, and adverse pulmonary reactions in humans^65^. Consistent with these reports, the impairment of epithelial barrier function observed in hBECs following treatment with AddaS03 spiked with CPC but not with AddaS03 alone, further supports the utility of this in vitro airway epithelial model as a relevant platform for evaluating the safety of candidate mucosal adjuvant formulations.

Nasal and bronchial airway epithelia are integral components of the respiratory epithelium. Despite their different embryonic origin, they consist of similar cell types and exhibit comparable pseudostratified morphology^66,67^. Moreover, both nasal and bronchial epithelia employ similar innate defense mechanisms, including the production of antimicrobial compounds and expression of Toll-like Receptors (TLRs) and NOD–like receptors, which trigger the release of proinflammatory cytokines and chemokines to recruit immune cells such as neutrophils and T cells to the site of infection^68,69^. In our study, no major differences were observed between differentiated hBEC and hNEC cultures in terms of goblet cell frequency or cilia expression. However, the hNEC culture exhibited greater epithelial thickness compared with the hBEC culture. Notably, the loss of transepithelial electrical resistance and of cell viability was less pronounced in hNECs, and only minor alterations in membrane ZO-1 expression were observed in hNECs compared with hBEC in response to the same NE01 doses. The attenuated responsiveness of the intranasal mucosa was suggested to be mediated by its overall immunosuppressive or tolerogenic microenvironment^70^. Our findings suggest that, in addition to the enrichment of CD4⁺CD25⁻ regulatory T cells in the nasal mucosa, previously implicated in maintaining nasal immune tolerance^71^, differences in tissue thickness and possibly intrinsic signaling properties of nasal epithelial cells in response to external stimuli, may also contribute to the immunosuppressive status of the nasal mucosa.

Recent transcriptome studies of airway tissue samples have revealed differences between the nasal and bronchial epithelia, including variations in ciliated and secretory cell composition, as well as the expression of distinct transcriptional regulatory proteins^45,72,73^. In addition, functional studies have demonstrated differences in receptor expression and susceptibility to infections with rhinoviruses, SARS-CoV-2, and avian and human influenza viruses^22,74,75^. The mechanisms underlying the differential sensitivity of hBEC and hNEC to the NE01 adjuvant remain to be elucidated and will be addressed in future studies. Nevertheless, data obtained from the ALI model are consistent with the well-established greater sensitivity of the LRT mucosa compared with the URT to excipients commonly used in mucosal vaccines, including stabilizers, surfactants, and adjuvants. Excipients that are well tolerated in the upper airway mucosa may elicit cytotoxic, proinflammatory, or irritant effects when deposited in the bronchial epithelium^76^.

A limitation of the study is the use of unpaired human bronchial and nasal epithelial cells from different donors, which precludes direct comparison of innate immune responses between the URT and LRT within the same individual. The NE01 concentrations used for in vitro treatments were scaled to account for the surface area of hBEC and hNEC cultures exposed to the adjuvant. These concentrations are not intended to replicate doses used in clinical studies but rather to enable assessment of local exposure, concentration-dependent effects, and thresholds for epithelial disruption. Accordingly, in vitro systems such as this ALI model do not allow for direct translation of dose levels to humans. Instead, they should be viewed as surrogate models that help identify potential safety signals and guide subsequent in vivo and clinical studies. While the ALI model provides an important contribution as a replacement for animal studies to monitor the health of the respiratory epithelium, the absence of white blood cells (e.g., neutrophils, monocytes, and dendritic cells) limits the overall analysis of the innate immune response induced by mucosal adjuvants.

In summary, the development of mucosal-targeting adjuvants has been greatly limited due to the difficulties of sampling mucosal tissues and the lack of biomarkers with which to assess mucosal immune responses and safety. We provided proof-of-concept supporting the use of in vitro differentiated airway ALI systems for early screening of candidate mucosal adjuvants to select optimal formulations devoid of cellular toxicity and proinflammatory responses. Our study demonstrates concentration-dependent effects of NE01 on mucosal epithelial barrier function and inflammatory responses, as well as differential impacts on hBEC and hNEC. Collectively, these results support the use of ALI epithelial models as a human-relevant platform for the preclinical evaluation of mucosal adjuvants, offering a viable alternative to animal testing and aligning with the principles of the 3Rs (Replacement, Reduction, and Refinement).

## Methods

### In vitro human air–liquid interface (ALI) airway tissue model

#### Expansion of primary human bronchial epithelial cells

Human ALI airway tissue model of bronchial epithelial cells was established as described previously^77^. Cryopreserved primary human bronchial epithelial cells (hBECs) from two healthy donors (The Cystic Fibrosis Center Tissue Procurement and Cell Culture Core, University of North Carolina, Chapel Hill, NC, USA^78^) were first expanded in complete PneumaCult Ex Plus Basal medium (Complete Basal medium, StemCell Technologies) supplemented with 0.5% hydrocortisone (StemCell) in T75 flask (Corning). When cells reached 90% confluence, they were harvested using Accutase (Innovative Cell Technologies), which was inactivated by Complete Basal medium. Expanded hBECs were frozen in FBS containing 10% DMSO and stored at −196 °C until further use.

#### Expansion of primary nasal epithelial cells using feeder cells

Human primary nasal epithelial cells were expanded using irradiated feeder fibroblasts^79^. Cryopreserved irradiated 3T3-J2 mouse fibroblasts (StemCell) were thawed and were resuspended in compete conditional reprogramming (CR) medium (StemCell) supplemented with 8.6 ng/mL of Cholera Toxin (MilliporeSigma). Cryopreserved human Nasal Epithelial cells (hNECs), isolated from the middle turbinate (The Cystic Fibrosis Center), from two healthy donors were thawed, resuspended in complete CR medium, were mixed with feeder cells at a ratio of ~1 million NECs and 2 to 3 million feeder cells in 10 mL of CR medium, and were seeded on a 10-cm^2^ dish precoated with PureCol 100 (Advance Biomaterials). The next day, the medium was aspirated to remove all dead cells and was replaced with fresh CR medium. On Days 3 to 4, CR medium was removed from the dishes, replaced with PBS (Ca, Mg-free), and feeder cells were removed by pipetting up and down. Expanded hNECs were harvested using Accutase and were frozen.

#### Seeding of hBECs and of hNECs on inserts

Frozen hBECs were thawed in Complete Basal medium and were expanded in a T75 flask. Frozen hNECs were expanded in CR medium on a 10-cm^2^ dish precoated with PureCol 100. Once cells reached about 50% confluency, they were harvested using Accutase and were seeded at a density of 1 × 10^5^ cells in 200 μL of Complete Basal medium or CR medium, per insert on the apical side of 0.4-μm pore polyester membrane inserts diameter 6.5 mm (0.33 cm^2^ cell-growth area, Costar) precoated with human placenta collagen Type IV (MilliporeSigma) and placed into wells of 24-well plate (Avantor). Five hundred microliters of the Complete Basal medium were added to the basolateral compartment. After 48- to 72-h, confluent monolayers were airlifted by aspirating the medium from both compartments, and 500 μL of complete PneumaCult ALI medium (StemCell) supplemented with 2.5 μL of Hydrocortisone and 1 μL of Heparin Solution 0.2% (StemCell) were added to the basolateral compartment only. The cell culture medium in the basolateral compartment was refreshed every other day for 4 weeks, by which time the cultures had become fully differentiated as determined by the presence of ciliated cells and goblet cells. From week 2 post airlift, the apical side of the cells was washed once a week using 200 μL PBS (Ca and Mg-free) to remove excess mucus. Cells were kept in a humidified incubator at 37 °C and 5% CO_2_.

#### Demographics of hBEC and hNEC donors used in the study

hBECs were obtained from two donors: Donor 1, a 70-year-old male, and Donor 2, a 65-year-old female. hNECs were obtained from two donors: Donor 3, a 47-year-old male, and Donor 4, a 58-year-old female. Each experiment was performed in duplicate using cells from two independent donors. Results shown in Figs. 1, 2, and 4 were generated using hBECs from Donor 1, whereas Fig. 3 presents data obtained using hBECs from Donor 2. Experiments shown in Figs. 5 and 6 were performed using hNECs from Donor 3; Fig. 7 shows results obtained with cells from Donor 2 and Donor 4.

### Cell treatments

NE01 adjuvant stock used in the study is a 60% formulation of the W_80_5EC nanoemulsion (60% W_80_5EC) containing 37.6% soybean oil, Tween 80 (3.5%), cetylpyridinium chloride (0.64%), and ethanol (4%) in water^28^. NE01 adjuvant and AddaS03 adjuvant containing 5% (v/v) squalene oil (InVivogen), were diluted in Complete Basal Medium to oil concentrations of equal or below 0.4%. In some experiments, cells were treated with NE01 adjuvant (diluted to 0.4% oil) mixed at a 1:2 ratio with proprietary-formulation buffer containing rHA antigen from the H5N1 A/Indonesia/05/05 strain of influenza virus (rH5, 0.6 µg^32^) or rH5 alone (0.6 µg per cell culture). In some experiments, cells were treated with AddaS03 adjuvant spiked with cetylpyridinium chloride (CPC, MilliporeSigma). Mucin was removed from the cell surface prior to cell treatment by adding PBS (Ca, Mg-free) to the apical side of hBEC or hNEC for 30 min at 37 °C, followed by PBS removal and then additional incubation for 5 min with fresh PBS. PBS was removed, and NE01, NE01/rHA, rHA alone, AddaS03, or AddaS03 with CPC in Complete Basal medium were added to the apical chambers in 100 µL volumes. After 1-h incubation at 37 °C and 5% CO_2_, treatments were removed, and the apical cell surface was exposed to air (returned to ALI).

### Trans-epithelial electrical resistance (TEER)

Changes in tissue permeability were evaluated by measuring the trans-epithelial electrical resistance (TEER) using an EVOM2 epithelial Volt–Ohmmeter (World Precision Instruments) and STX-2 chopstick electrode. Cell cultures were equilibrated to room temperature. Two hundred microliters of PBS (Ca, Mg-free) were added to the apical compartment of the culture inserts. Raw resistance values (Ω) were corrected by subtracting the resistance of blank inserts and multiplying by the membrane surface area (cm^2^) to obtain TEER values expressed as Ω·cm².

TEER measurements were performed immediately prior to each experiment on two inserts per batch to confirm TEER values of 300 to 500 Ω·cm² and 200 to 300 Ω·cm², in fully differentiated hBEC and hNEC, respectively.

### Cell viability assay

Viability of hBECs and hNECs post treatments was evaluated by the assessment of lactate dehydrogenase (LDH) release in hBEC and hNEC cell culture supernatants (collected from the basolateral chamber) using the CytoTox 96 Non-Radioactive Cytotoxicity Assay (Promega) following the manufacturer’s instructions. In brief, 50 µL of cell culture medium from the basolateral chamber of each test insert was transferred to a 96-well assay plate. For positive control, 100 µL of 10X Lysis buffer was added to untreated hBECs or hNECs 45 min before the assay to obtain complete cell lysis and 50 µL of the cell lysate was transferred to the 96-well assay plate. An equal volume of CytoTox 96 reagent was added to all test and control samples. After 30 min incubation, the stop solution was added, and the absorbance signal was measured at OD 490 nm in a Synergy2 Multi-Mode plate reader (BioTek Instruments). The LDH in each insert was quantified using technical replicates (*n* = 2) in the assay. The LDH was calculated as follows: % LDH = 100 × [experimental LDH release (OD_490_)/maximum LDH release (OD_490_)].

### Apoptosis assay (Caspase 3/7)

Apoptosis in hBECs and hNECs was measured using a Caspase-Glo 3/7 3D kit following the manufacturer’s instructions (Promega). Two hundred microliters of the Caspase-Glo reagent were added to the apical surface of cells. Plates were gently mixed on an orbital shaker for 30 s and were further incubated at room temperature for 60 min. One hundred microliters of cell lysate were transferred to a 96-well white-walled microplate (Corning) prior to reading. Luminescence measurements were obtained using the Synergy2 reader. Caspase 3/7 activity in cell-free inserts was used to measure background luminescence. A purified Caspase 3 enzyme (Enzo Biochem Inc.) diluted from 100 U/well to 0.1 U/well was used as the assay positive control in each assay.

### Immunofluorescence and confocal microscopy

#### ZO-1 staining

Cells were fixed in 100% methanol (added to upper and lower chambers) at −20 °C for 30 min, washed with PBS and were blocked in Blocking buffer containing 1% bovine serum albumin (BSA), 10% normal goat serum (Invitrogen), 0.05% Tween 20, and 0.1% Triton X-100 for 1 h. Cells were stained with mouse monoclonal or rabbit polyclonal anti-human ZO-1 antibody at 10 µg/mL (Invitrogen) in blocking buffer mixed 1:1 with PBS for 1 h at room temperature (RT). Membranes were washed three times with PBS before adding the goat anti-mouse IgG or goat anti-rabbit IgG secondary antibody conjugated to Alexa Fluor 488 at 1 µg/mL (Invitrogen) for 30 min.

#### MUC5A and β-tubulin staining

Cells were fixed in 4% paraformaldehyde in DPBS with Ca and Mg for 30 min at RT, permeabilized with 0.2% Triton X-100 in PBS for 15 min at RT and blocked with 1% BSA and 10% normal goat serum. Cells were stained with rabbit anti-β-tubulin Alexa Fluor 555 antibody (1:100) (Cell Signaling), a marker of ciliated cells for 40 min in Blocking buffer mixed 1:1 with PBS or with mouse anti-MUC5AC monoclonal antibody (marker of goblet cells) (Invitrogen) (1:100) for 1 h followed by goat anti-mouse IgG Alexa Fluor 488 secondary antibody at 1 µg/mL (Invitrogen).

The membranes were excised using a scalpel and mounted on glass slides using DAPI mounting solution (Vectashield, Vector Labs). Slides were left to dry overnight, covered with cover slips, and the edges were sealed with clear nail polish.

Images of cells stained with anti-ZO-1 antibodies were acquired using an Eclipse TS2R-FL microscope with epifluorescence (Nikon) and processed using Nikon NIS-Elements Software.

Images of hBECs and hNECs stained with anti-MUC5AC and anti-β-tubulin antibodies were acquired using a Leica TCS SP8 confocal laser scanning microscope (Leica Microsystems, Germany) equipped with ultraviolet and white light lasers. Images were captured with a 40× oil-immersion objective lens (NA 1.3). At least three z-stack images were collected at 1 µm intervals, generating 30–50 optical sections per sample. Image stacks were saved in Leica image (LIF) format for subsequent analysis. Three-dimensional image processing and quantitative analyses were performed using Imaris software (Bitplane, Oxford Instruments).

### Measurements of IL-8 and MMP9 by enzyme-linked immunosorbent assay (ELISA)

IL-8 cytokine and MMP9 metalloproteinase were measured in the hNECs and hBECs cell culture supernatant using human Quantikine enzyme-linked immunosorbent assay (ELISA) kits (R&D Systems). IL-8 and MMP9 in each cell culture were quantified using technical replicates (*n* = 2) in the respective assay. All plates were read by a Synergy2 reader.

### Statistical analysis

Statistical analysis was performed using GraphPad Prism software, version 10.4.1. Data were analyzed with an unpaired two-tailed Student’s *t*-test, and significance of differences was determined by two-way Anova with Sidak’s multiple comparisons test, by one-way Anova with Dunnett’s multiple comparisons test, or an unpaired, two-tailed nonparametric Mann-Whitney *U* test. *P* values of ≤0.05 were considered statistically significant. Sample sizes for each experimental condition and details of the statistical analyses are provided in the figure legends.

### Usage of animals in the study

No animals or animal-derived cells were used in the experiments.

## Supplementary information

Supplementary Information

## Data Availability

Data were provided within the manuscript or supplementary information files.

## References

[1] Zhang, Z. et al. Mucosal immunity and vaccination strategies: current insights and future perspectives. *Mol. Biomed.* **6**, 57 (2025). 40830509 10.1186/s43556-025-00301-7 PMC12364801

[2] Jung, J. et al. Transmission and infectious SARS-CoV-2 shedding kinetics in vaccinated and unvaccinated individuals. *JAMA Netw. Open* **5**, e2213606 (2022). 35608859 10.1001/jamanetworkopen.2022.13606 PMC9131744

[3] Puhach, O. et al. Infectious viral load in unvaccinated and vaccinated individuals infected with ancestral, Delta or Omicron SARS-CoV-2. *Nat. Med.* **28**, 1491–1500 (2022). 35395151 10.1038/s41591-022-01816-0

[4] Singanayagam, A. et al. Community transmission and viral load kinetics of the SARS-CoV-2 delta (B.1.617.2) variant in vaccinated and unvaccinated individuals in the UK: a prospective, longitudinal, cohort study. *Lancet Infect. Dis.* **22**, 183–195 (2022). 34756186 10.1016/S1473-3099(21)00648-4 PMC8554486

[5] Lavelle, E. C. & Ward, R. W. Mucosal vaccines - fortifying the frontiers. *Nat. Rev. Immunol.* **22**, 236–250 (2022). 34312520 10.1038/s41577-021-00583-2 PMC8312369

[6] Macpherson, A. J., McCoy, K. D., Johansen, F. E. & Brandtzaeg, P. The immune geography of IgA induction and function. *Mucosal Immunol.* **1**, 11–22 (2008). 19079156 10.1038/mi.2007.6

[7] Sinha, D., Yaugel-Novoa, M., Waeckel, L., Paul, S. & Longet, S. Unmasking the potential of secretory IgA and its pivotal role in protection from respiratory viruses. *Antivir. Res.* **223**, 105823 (2024). 38331200 10.1016/j.antiviral.2024.105823

[8] Mao, T. et al. Unadjuvanted intranasal spike vaccine elicits protective mucosal immunity against sarbecoviruses. *Science* **378**, eabo2523 (2022). 36302057 10.1126/science.abo2523 PMC9798903

[9] Boyaka, P. N. Inducing mucosal IgA: A challenge for vaccine adjuvants and delivery systems. *J. Immunol.* **199**, 9–16 (2017). 28630108 10.4049/jimmunol.1601775 PMC5719502

[10] Li, J. et al. Advances and prospects of respiratory mucosal vaccines: mechanisms, technologies, and clinical applications. *NPJ Vaccines* **10**, 230 (2025). 41224752 10.1038/s41541-025-01280-0 PMC12612081

[11] Zeng, L. Mucosal adjuvants: opportunities and challenges. *Hum. Vaccin. Immunother.* **12**, 2456–2458 (2016). 27159278 10.1080/21645515.2016.1181236 PMC5027714

[12] Maciel, M. Jr. et al. Evaluation of the reactogenicity, adjuvanticity and antigenicity of LT(R192G) and LT(R192G/L211A) by intradermal immunization in mice. *PLoS ONE* **14**, e0224073 (2019). 31682624 10.1371/journal.pone.0224073 PMC6827915

[13] Seok, J. et al. Genomic responses in mouse models poorly mimic human inflammatory diseases. *Proc. Natl. Acad. Sci. USA* **110**, 3507–3512 (2013). 23401516 10.1073/pnas.1222878110 PMC3587220

[14] Zeiss, C. J. & Brayton, C. F. Immune responses to the real world. *Lab Anim.* **47**, 13–14 (2018). 10.1038/laban.1384 29297473

[15] Grainger, C. I., Greenwell, L. L., Lockley, D. J., Martin, G. P. & Forbes, B. Culture of Calu-3 cells at the air interface provides a representative model of the airway epithelial barrier. *Pharm. Res.* **23**, 1482–1490 (2006). 16779708 10.1007/s11095-006-0255-0

[16] Silva, S., Bicker, J., Falcão, A. & Fortuna, A. Air-liquid interface (ALI) impact on different respiratory cell cultures. *Eur. J. Pharm. Biopharm.* **184**, 62–82 (2023). 36696943 10.1016/j.ejpb.2023.01.013

[17] Kesimer, M. et al. Tracheobronchial air-liquid interface cell culture: a model for innate mucosal defense of the upper airways?. *Am. J. Physiol. Lung Cell. Mol. Physiol.* **296**, L92–L100 (2009). 18931053 10.1152/ajplung.90388.2008 PMC2636953

[18] Prytherch, Z. et al. Tissue-specific stem cell differentiation in an in vitro airway model. *Macromol. Biosci.* **11**, 1467–1477 (2011). 21994115 10.1002/mabi.201100181

[19] Fulcher, M. L. & Randell, S. H. Human nasal and tracheo-bronchial respiratory epithelial cell culture. *Methods Mol. Biol.* **945**, 109–121 (2013). 23097104 10.1007/978-1-62703-125-7_8

[20] Rayner, R. E., Makena, P., Prasad, G. L. & Cormet-Boyaka, E. Optimization of normal human bronchial epithelial (NHBE) cell 3D cultures for in vitro lung model studies. *Sci. Rep.* **9**, 500 (2019). 30679531 10.1038/s41598-018-36735-z PMC6346027

[21] Zhao, C. et al. Activation of STAT3-mediated ciliated cell survival protects against severe infection by respiratory syncytial virus. *J. Clin. Invest.* **134**, e183978 (2024). 39484716 10.1172/JCI183978 PMC11527452

[22] Hou, Y. J. et al. SARS-CoV-2 reverse genetics reveals a variable infection gradient in the respiratory tract. *Cell* **182**, 429–446 e414 (2020). 32526206 10.1016/j.cell.2020.05.042 PMC7250779

[23] Liang, K. et al. Initiator cell death event induced by SARS-CoV-2 in the human airway epithelium. *Sci. Immunol.* **9**, eadn0178 (2024). 38996010 10.1126/sciimmunol.adn0178 PMC11970318

[24] Stolting, H. et al. Distinct airway epithelial immune responses after infection with SARS-CoV-2 compared to H1N1. *Mucosal Immunol.* **15**, 952–963 (2022). 35840680 10.1038/s41385-022-00545-4 PMC9284972

[25] Major, J. et al. Type I and III interferons disrupt lung epithelial repair during recovery from viral infection. *Science* **369**, 712–717 (2020). 32527928 10.1126/science.abc2061 PMC7292500

[26] Song, B. M., Kang, Y. M., Kim, H. S. & Seo, S. H. Induction of inflammatory cytokines and toll-like receptors in human normal respiratory epithelial cells infected with seasonal H1N1, 2009 pandemic H1N1, seasonal H3N2, and highly pathogenic H5N1 influenza virus. *Viral Immunol.* **24**, 179–187 (2011). 21668359 10.1089/vim.2010.0125

[27] Forero, A. et al. Evaluation of the innate immune responses to influenza and live-attenuated influenza vaccine infection in primary differentiated human nasal epithelial cells. *Vaccine* **35**, 6112–6121 (2017). 28967519 10.1016/j.vaccine.2017.09.058 PMC5647870

[28] Makidon, P. E. et al. Pre-clinical evaluation of a novel nanoemulsion-based hepatitis B mucosal vaccine. *PLoS ONE* **3**, e2954 (2008). 18698426 10.1371/journal.pone.0002954 PMC2496893

[29] Hamouda, T., Sutcliffe, J. A., Ciotti, S. & Baker, J. R. Jr. Intranasal immunization of ferrets with commercial trivalent influenza vaccines formulated in a nanoemulsion-based adjuvant. *Clin. Vaccin. Immunol.* **18**, 1167–1175 (2011). 10.1128/CVI.00035-11 PMC3147313 21543588

[30] Stanberry, L. R. et al. Safety and immunogenicity of a novel nanoemulsion mucosal adjuvant W805EC combined with approved seasonal influenza antigens. *Vaccine* **30**, 307–316 (2012). 22079079 10.1016/j.vaccine.2011.10.094

[31] Das, S. C. et al. Nanoemulsion W805EC improves immune responses upon intranasal delivery of an inactivated pandemic H1N1 influenza vaccine. *Vaccine* **30**, 6871–6877 (2012). 22989689 10.1016/j.vaccine.2012.09.007 PMC3488283

[32] Wang, S. H. et al. Recombinant H5 hemagglutinin adjuvanted with nanoemulsion protects ferrets against pathogenic avian influenza virus challenge. *Vaccine* **37**, 1591–1600 (2019). 30795941 10.1016/j.vaccine.2019.02.002

[33] Smith, D., Streatfield, S. J., Acosta, H., Ganesan, S. & Fattom, A. A nanoemulsion-adjuvanted intranasal H5N1 influenza vaccine protects ferrets against homologous and heterologous H5N1 lethal challenge. *Vaccine* **37**, 6162–6170 (2019). 31495593 10.1016/j.vaccine.2019.08.071

[34] Deming, M. E. et al. An intranasal adjuvanted, recombinant influenza A/H5 vaccine primes against diverse H5N1 clades: a phase I trial. *Nat. Commun.* **16**, 9321 (2025). 41198655 10.1038/s41467-025-64686-3 PMC12592354

[35] Roy, C. J. et al. Aerosolized adenovirus-vectored vaccine as an alternative vaccine delivery method. *Respir. Res.* **12**, 153 (2011). 22103776 10.1186/1465-9921-12-153 PMC3287261

[36] Xu, H., Cai, L., Hufnagel, S. & Cui, Z. Intranasal vaccine: factors to consider in research and development. *Int. J. Pharm.* **609**, 121180 (2021). 34637935 10.1016/j.ijpharm.2021.121180

[37] Jeyanathan, M. et al. Induction of lung mucosal immunity by a next-generation inhaled aerosol COVID-19 vaccine: an open-label, multi-arm phase 1 clinical trial. *Nat. Commun.* **16**, 6000 (2025). 40603330 10.1038/s41467-025-60726-0 PMC12223143

[38] Huvenne, W. et al. Different regulation of cigarette smoke induced inflammation in upper versus lower airways. *Respir. Res.* **11**, 100 (2010). 20650015 10.1186/1465-9921-11-100 PMC2915966

[39] Sahakijpijarn, S., Smyth, H. D. C., Miller, D. P. & Weers, J. G. Post-inhalation cough with therapeutic aerosols: formulation considerations. *Adv. Drug Deliv. Rev.* **165-166**, 127–141 (2020). 32417367 10.1016/j.addr.2020.05.003

[40] Haschek, W. M., Witschi, H. R. & Nikula, K. J. in *Handbook of Toxicologic Pathology* (*Second Edition*) (eds Haschek, W. M., Rousseaux, C. G. & Wallig, M. A.) (Academic Press, 2002).

[41] Liu, Y., Johnson, M. R., Matida, E. A., Kherani, S. & Marsan, J. Creation of a standardized geometry of the human nasal cavity. *J. Appl. Physiol.* **106**, 784–795 (2009). 19131483 10.1152/japplphysiol.90376.2008

[42] Marshall, L. J., Perks, B., Ferkol, T. & Shute, J. K. IL-8 released constitutively by primary bronchial epithelial cells in culture forms an inactive complex with secretory component. *J. Immunol.* **167**, 2816–2823 (2001). 11509627 10.4049/jimmunol.167.5.2816

[43] Atkinson, J. J. & Senior, R. M. Matrix metalloproteinase-9 in lung remodeling. *Am. J. Respir. Cell Mol. Biol.* **28**, 12–24 (2003). 12495928 10.1165/rcmb.2002-0166TR

[44] Wolosowicz, M., Prokopiuk, S. & Kaminski, T. W. Matrix Metalloproteinase-9 (MMP-9) as a therapeutic target: insights into molecular pathways and clinical applications. *Pharmaceutics* **17**, 1425 (2025). 41304763 10.3390/pharmaceutics17111425 PMC12655286

[45] Rodenburg, L. W. et al. Exploring intrinsic variability between cultured nasal and bronchial epithelia in cystic fibrosis. *Sci. Rep.* **13**, 18573 (2023). 37903789 10.1038/s41598-023-45201-4 PMC10616285

[46] Horowitz, A., Chanez-Paredes, S. D., Haest, X. & Turner, J. R. Paracellular permeability and tight junction regulation in gut health and disease. *Nat. Rev. Gastroenterol. Hepatol.* **20**, 417–432 (2023). 37186118 10.1038/s41575-023-00766-3 PMC10127193

[47] Otani, T. & Furuse, M. Tight junction structure and function revisited. *Trends Cell Biol.* **30**, 1014 (2020). 32891490 10.1016/j.tcb.2020.08.004

[48] Gonzalez-Mariscal, L., Betanzos, A., Nava, P. & Jaramillo, B. E. Tight junction proteins. *Prog. Biophys. Mol. Biol.* **81**, 1–44 (2003). 12475568 10.1016/s0079-6107(02)00037-8

[49] Fanning, A. S. & Anderson, J. M. Zonula occludens-1 and -2 are cytosolic scaffolds that regulate the assembly of cellular junctions. *Ann. N. Y Acad. Sci.* **1165**, 113–120 (2009). 19538295 10.1111/j.1749-6632.2009.04440.x PMC3759978

[50] Umeda, K. et al. ZO-1 and ZO-2 independently determine where claudins are polymerized in tight-junction strand formation. *Cell* **126**, 741–754 (2006). 16923393 10.1016/j.cell.2006.06.043

[51] Vermeer, P. D. et al. MMP9 modulates tight junction integrity and cell viability in human airway epithelia. *Am. J. Physiol. Lung Cell. Mol. Physiol.* **296**, L751–L762 (2009). 19270179 10.1152/ajplung.90578.2008 PMC2681350

[52] Myc, A. et al. Nanoemulsion nasal adjuvant W(8)(0)5EC induces dendritic cell engulfment of antigen-primed epithelial cells. *Vaccine* **31**, 1072–1079 (2013). 23273511 10.1016/j.vaccine.2012.12.033 PMC3556236

[53] Bielinska, A. U. et al. Mucosal immunization with a novel nanoemulsion-based recombinant anthrax protective antigen vaccine protects against *Bacillus anthracis* spore challenge. *Infect. Immun.* **75**, 4020–4029 (2007). 17502384 10.1128/IAI.00070-07 PMC1952013

[54] Bielinska, A. U. et al. Nasal immunization with a recombinant HIV gp120 and nanoemulsion adjuvant produces Th1 polarized responses and neutralizing antibodies to primary HIV type 1 isolates. *AIDS Res. Hum. Retroviruses* **24**, 271–281 (2008). 18260780 10.1089/aid.2007.0148

[55] Bielinska, A. U. et al. Distinct pathways of humoral and cellular immunity induced with the mucosal administration of a nanoemulsion adjuvant. *J. Immunol.* **192**, 2722–2733 (2014). 24532579 10.4049/jimmunol.1301424 PMC3948110

[56] Mao, X. et al. Cetylpyridinium chloride: mechanism of action, antimicrobial efficacy in biofilms, and potential risks of resistance. *Antimicrob. Agents Chemother.* **64**, 10–1128 (2020). 10.1128/AAC.00576-20 PMC7526810 32513792

[57] Wong, P. T. et al. Formulation and characterization of nanoemulsion intranasal adjuvants: effects of surfactant composition on mucoadhesion and immunogenicity. *Mol. Pharm.* **11**, 531–544 (2014). 24320221 10.1021/mp4005029 PMC4031264

[58] Illum, L. Nasal drug delivery–possibilities, problems and solutions. *J. Control Release* **87**, 187–198 (2003). 12618035 10.1016/s0168-3659(02)00363-2

[59] Kanno, S. et al. Benzalkonium chloride and cetylpyridinium chloride induce apoptosis in human lung epithelial cells and alter surface activity of pulmonary surfactant monolayers. *Chem. Biol. Interact.* **317**, 108962 (2020). 31982400 10.1016/j.cbi.2020.108962

[60] Yegin, Y., Oh, J. K., Akbulut, M. & Taylor, T. Cetylpyridinium chloride produces increased zeta-potential on *Salmonella* Typhimurium cells, a mechanism of the pathogen’s inactivation. *npj Sci. Food* **3**, 21 (2019). 31633036 10.1038/s41538-019-0052-x PMC6795798

[61] Sharma, V. et al. Protein-surfactant interactions: a multitechnique approach on the effect of co-solvents over bovine serum albumin (BSA)-cetyl pyridinium chloride (CPC) system. *Chem. Phys. Lett.* **747**, 137349 (2020).

[62] Bernstein, I. L. Is the use of benzalkonium chloride as a preservative for nasal formulations a safety concern? A cautionary note based on compromised mucociliary transport. *J. Allergy Clin. Immunol.* **105**, 39–44 (2000). 10629450 10.1016/s0091-6749(00)90175-1

[63] Larsen, S. T., Verder, H. & Nielsen, G. D. Airway effects of inhaled quaternary ammonium compounds in mice. *Basic Clin. Pharm. Toxicol.* **110**, 537–543 (2012). 10.1111/j.1742-7843.2011.00851.x 22188809

[64] Kwon, D. et al. Evaluation of pulmonary toxicity of benzalkonium chloride and triethylene glycol mixtures using in vitro and in vivo systems. *Environ. Toxicol.* **34**, 561–572 (2019). 30786124 10.1002/tox.22722 PMC6594094

[65] George, M., Joshi, S. V., Concepcion, E. & Lee, H. Paradoxical bronchospasm from benzalkonium chloride (BAC) preservative in albuterol nebulizer solution in a patient with acute severe asthma. A case report and literature review of airway effects of BAC. *Respir. Med. Case Rep.* **21**, 39–41 (2017). 28377880 10.1016/j.rmcr.2017.03.005 PMC5369872

[66] Herriges, M. & Morrisey, E. E. Lung development: orchestrating the generation and regeneration of a complex organ. *Development* **141**, 502–513 (2014). 24449833 10.1242/dev.098186 PMC3899811

[67] Widdicombe, J. H. Early studies on the surface epithelium of mammalian airways. *Am. J. Physiol. Lung Cell. Mol. Physiol.* **317**, L486–L495 (2019). 31313615 10.1152/ajplung.00240.2019

[68] Parker, D. & Prince, A. Innate immunity in the respiratory epithelium. *Am. J. Respir. Cell Mol. Biol.* **45**, 189–201 (2011). 21330463 10.1165/rcmb.2011-0011RT PMC3175551

[69] Ioannidis, I., Ye, F., McNally, B., Willette, M. & Flano, E. Toll-like receptor expression and induction of type I and type III interferons in primary airway epithelial cells. *J. Virol.* **87**, 3261–3270 (2013). 23302870 10.1128/JVI.01956-12 PMC3592129

[70] Golebski, K. et al. FcgammaRIII stimulation breaks the tolerance of human nasal epithelial cells to bacteria through cross-talk with TLR4. *Mucosal Immunol.* **12**, 425–433 (2019). 30664707 10.1038/s41385-018-0129-x

[71] Unger, W. W. et al. Nasal tolerance induces antigen-specific CD4+CD25- regulatory T cells that can transfer their regulatory capacity to naive CD4+ T cells. *Int. Immunol.* **15**, 731–739 (2003). 12750357 10.1093/intimm/dxg069

[72] Imkamp, K. et al. Gene network approach reveals co-expression patterns in nasal and bronchial epithelium. *Sci. Rep.* **9**, 15835 (2019). 31676779 10.1038/s41598-019-50963-x PMC6825243

[73] Vieira Braga, F. A. et al. A cellular census of human lungs identifies novel cell states in health and in asthma. *Nat. Med.* **25**, 1153–1163 (2019). 31209336 10.1038/s41591-019-0468-5

[74] Shinya, K. et al. Avian flu: influenza virus receptors in the human airway. *Nature* **440**, 435–436 (2006). 16554799 10.1038/440435a

[75] Alves, M. P. et al. Comparison of innate immune responses towards rhinovirus infection of primary nasal and bronchial epithelial cells. *Respirology* **21**, 304–312 (2016). 26611536 10.1111/resp.12692

[76] Frohlich, E. Better GRAS than sorry: excipient toxicity in pulmonary formulations. *Eur. J. Pharm. Biopharm.* **215**, 114814 (2025). 40714171 10.1016/j.ejpb.2025.114814

[77] Cao, X., Muskhelishvili, L., Latendresse, J., Richter, P. & Heflich, R. H. Evaluating the toxicity of cigarette whole smoke solutions in an air-liquid-interface human in vitro airway tissue model. *Toxicol. Sci.* **156**, 14–24 (2017). 28115645 10.1093/toxsci/kfw239

[78] Fulcher, M. L., Gabriel, S., Burns, K. A., Yankaskas, J. R. & Randell, S. H. Well-differentiated human airway epithelial cell cultures. *Methods Mol. Med.* **107**, 183–206 (2005). 15492373 10.1385/1-59259-861-7:183

[79] Gentzsch, M. et al. Pharmacological rescue of conditionally reprogrammed cystic fibrosis bronchial epithelial cells. *Am. J. Respir. Cell Mol. Biol.* **56**, 568–574 (2017). 27983869 10.1165/rcmb.2016-0276MA PMC5449492

