# Supplementary material for: Development of in vitro airway epithelial model to assess immune response and safety of mucosal adjuvants
Source: NPJ Vaccines. 2026 Apr 22;11:124. doi: 10.1038/s41541-026-01459-z (PMC13287757; doi:10.1038/s41541-026-01459-z)
Supplement: Supplementary file 1 — Supplementary Information [file 41541_2026_1459_MOESM1_ESM.pdf]

Supplementary Table 1 Quantification of MUC5AC-positive cells in ALI-differentiated human bronchial and nasal epithelial cultures

**Cell type**	**Microscopic field**	**Number of nuclei**	**Number of MUC5AC Positive cells**	**Frequency of MUC5AC positive cells (%)**
hBEC	hBEC1	2468	19	0.770
	hBEC2	2411	22	0.912
	hBEC3	2892	15	0.519
hNEC	hNEC1	2817	18	0.639
	hNEC2	2736	14	0.512
	hNEC3	2673	10	0.374

ALI-differentiated hBECs and hNECs were obtained from a single donor per cell type and stained with anti-MUC5AC antibodies. For each culture, three representative microscopic fields were analyzed using Imaris software to quantify total nuclei and MUC5AC-positive cells. The frequency of MUC5AC-positive cells is expressed as a percentage of total nuclei within each microscopic field. Data represent technical replicates from three microscopic fields per culture.
